# World Arthritis Day 2018 and National Mental Illness Awareness Week

**DOI:** 10.15585/mmwr.mm6739a1

**Published:** 2018-10-05

World Arthritis Day* is October 12, 2018, and National Mental Illness Awareness Week^†^ is October 7–13, 2018. World Arthritis Day encourages organizations and individuals to work toward increasing awareness about arthritis and other rheumatic conditions worldwide. National Mental Illness Awareness Week seeks to educate the public, combat stigma, and provide support to those affected by mental illness.

A report in this issue found that adults with arthritis had higher prevalences of symptoms of anxiety (22.5%) and depression (12.1%) compared with adults without arthritis (*1*). Community-delivered self-management educational programs, such as the Chronic Disease Self-Management Program,^§^ can increase self-efficacy (confidence) and physical activity (e.g., walking), improve self-rated health, and reduce depression, fatigue, and pain (*2*). CDC works with national and state partners to disseminate these educational programs in communities.

## Footnotes

* https://www.eular.org/what_we_do_dont_delay_connect_today_2018.cfm.

† https://www.nami.org/Get-Involved/Raise-Awareness/Awareness-Events/Mental-Illness-Awareness-Week.

§ https://www.cdc.gov/arthritis/marketing-support/1-2-3-approach/docs/pdf/provider_fact_sheet_cdsmp.pdf.
